# Anthrax

**Published:** 1895-10

**Authors:** 


### ANTHRAX.

*New York.*—In eight counties.

*Tennessee.*—Eastern and Middle sections; State Veterinarian Rayen directing the work looking to its control.

